# LED lighting as a solution for turf shading: insights from a systemic assessment of *Seashore paspalum* and *Zoysia japonica*

**DOI:** 10.3389/fpls.2026.1936506

**Published:** 2026-09-04

**Authors:** Weihao Dou, Wan Geng, Weisen Sun, Jie Xu, Wenchao Zhang, Guilong Song, Yufeng Chen, Liebao Han

**Affiliations:** School of Grassland Science, Beijing Forestry University, Beijing, China

**Keywords:** LED lighting, physiological response, root morphology, scene simulation, shade stress

## Abstract

**Introduction:**

The roof structures of professional enclosed stadiums often cause shading problems on the turf, posing a major challenge to maintaining turf quality and efficient management. Artificial lighting has been applied to address this issue, particularly light-emitting diode (LED).

**Methods:**

To evaluate the efficacy of LED with a specific wavelength in alleviating the adverse effects of shade stress on turfgrasses, we conducted a systematic and multi-dimensional assessment of *Seashore paspalum* (*Paspalum vaginatum* Sw.) and *Zoysia japonica* (*Zoysia japonica* Steud.) turf under three light conditions: natural lighting, shading, and LED, within a stadium.

**Results:**

The results demonstrated that LED effectively alleviated shade-induced light limitation. It maintained turf normalized difference vegetation index (NDVI) and chlorophyll content at levels comparable to those under natural light. Furthermore, LED treatment improved physiological responses associated with shade stress, as indicated by enhanced cell membrane stability, reduced membrane lipid peroxidation, and altered antioxidant-related responses, including increased superoxide dismutase (SOD) activity and dynamic changes in peroxidase (POD) activity. Regarding root morphology, LED treatment reduced the shade-induced increases in specific root length (SRL) and specific root surface area (SRA), suggesting a modification of shade-induced root morphological responses. Principal component analysis (PCA) revealed distinct multivariate response patterns associated with different light environments. Correlation analysis further indicated significant associations among NDVI, chlorophyll content, and antioxidant-related traits across different light environments. These associations suggested that turf performance under LED lighting was related to concurrent variation in chlorophyll content and antioxidant-related traits.

**Discussion:**

In conclusion, applying LED lighting can effectively improve turf recovery-related traits and physiological status through improvements in canopy characteristics, chlorophyll status, antioxidant-related responses, and root morphological traits. This study demonstrated under a real stadium environment that specific LED lighting can serve as an effective supplemental light source for improving turf performance in shaded areas, providing practical guidance for stadium turf management.

## 1 Introduction

The quality of sports turf directly impacts the level of competition, athlete safety, and the spectator experience (Thanheiser et al., 2018; Çelik et al., 2019). Maintaining consistently high and uniform turf quality is a core objective of stadium operation and management (Curk et al., 2017). However, to enhance spectator experience and meet architectural aesthetics, modern stadiums commonly feature closed or semi-closed roof structures, which result in chronically insufficient natural light in certain areas of the pitch (Brito et al., 2023). As the primary energy source for plant photosynthesis, and shading has consequently emerged as the most significant limiting factor for healthy turf growth in such enclosed stadiums (Jiuxin and Liebao, 2022). Traditional maintenance practices such as fertilization and irrigation cannot make up for this fundamental energy deficit (Alexandre et al., 2018). Therefore, addressing the shade challenge of stadiums is urgently needed, as it has practical significance for ensuring the sustainable, high-quality maintenance and operation of stadium turf.

Currently, practical approaches to address shade challenge in sports stadiums primarily rely on selecting shade-tolerant turfgrass cultivars or employing traditional high-pressure sodium supplemental lighting (HPS) (Xie et al., 2020; Brito et al., 2023). However, the former is limited by the autologous physiological threshold of germplasm, while the latter suffers from issues such as high energy consumption, substantial heat radiation, and a spectral output that poorly matches plant physiological needs (Terfa et al., 2013; Dunne et al., 2017; Xu et al., 2022). In practical applications, these methods often have limited effects or are costly. In recent years, light-emitting diode (LED) technology has gained increasing attention in protected agriculture owing to its precise spectral control, high energy efficiency, and suitability for customized plant light management (Singh et al., 2015; Dunne et al., 2017). Existing research on LED have predominantly focused on crops and horticultural plants under controlled laboratory or greenhouse conditions (Singh et al., 2015). However, the effectiveness of supplemental LED lighting under actual stadium conditions remains difficult to evaluate because turfgrass is exposed to heterogeneous light environments caused by stadium architecture. Particularly under conditions that closely simulate the complex environments of real stadiums, where the integrated responses of turfgrass involving visual recovery, chlorophyll maintenance, antioxidant capacity, and root morphological traits require further investigation. This seriously restricts the scientific application and promotion of LED technology in professional stadiums maintenance practices.

Therefore, this study was designed to systematically evaluate the effectiveness of applying LED for shaded turf in professional sports stadiums. Based on the actual environment of a professional football stadium growth scene simulation, a full growing season experiment was conducted using two warm-season turfgrass species, *Seashore paspalum* (*Paspalum vaginatum* Sw.) and *Zoysia japonica* (*Zoysia japonica* Steud.), under different lighting conditions. The research focused on a comprehensive analysis of multidimensional physiological and growth responses, encompassing appearance, chlorophyll content, physiological stress tolerance, and root morphology. The findings were expected to improve the understanding of turfgrass responses to supplemental LED lighting under shaded stadium conditions and provide experimental evidence for the potential application of LED technology in enclosed sports turf management.

## 2 Methods

### 2.1 Experimental site and materials

The experiment was conducted from March to July 2024 within a professional enclosed stadium in Shanghai, China (31°14′ N, 121°32′ E). The tested plant materials were *Seashore paspalum* (cultivar Platinum) and *Zoysia japonica* (cultivar Lan Yin 3#). Before the formal experiment, turf modules were established and maintained from August 2023 to February 2024. After this preliminary maintenance period, all tested turf modules reached healthy and uniform growth conditions and were subsequently transferred into the stadium. To avoid affecting the normal use of the pitch for events, the experimental turf was laid on a stainless-steel movable turf module (1 m × 1 m, 15cm depth). The soil conditions were the same as those of the turf of the pitch. The irrigation and fertilization management measures for all the tested turf modules were always exactly the same.

Sampling and measurements were initiated in March 2024 and conducted once per month for five consecutive months. Each turf module was randomly sampled and measured once during each monthly sampling event. The monthly sampling and measurements were completed within the preceding week of each month, and the exact sampling dates were determined in advance according to the stadium operation schedule to minimize interference with normal stadium activities.

### 2.2 Experiment design

The experiment employed a design with three light treatments: (1) Natural light treatment (N): modules placed on the northern side of the stadium receiving ambient daylight without supplemental lighting or shading structures. (2) Shading treatment (S): modules placed on the southern side of the stadium, which receive no solar radiation or supplemental lighting. (3) LED light treatment (L): modules placed on the southern side, the shading degree was the same as that of the shade treatment, with supplemental LED lighting applied during nighttime hours (20:00 to 08:00, total 12 hours). This experimental layout was designed to simulate practical light environments and management practices in professional stadiums, where shaded areas with insufficient solar radiation are commonly managed with supplemental LED lighting to maintain turf quality. Three physically independent turf modules per species per treatment served as biological replicates (2 species×3 treatments×3 replicates, total 18 modules). Modules were treated as independent experimental units and spaced 30 cm apart to maintain separate light environments. Modules were randomly positioned within each designated area to minimize spatial heterogeneity. Due to stadium constraints, natural-light modules were located on the northern side, whereas shade and LED modules were located on the southern side, with no rotation during the experiment. A schematic diagram of the experimental turf modules layout is provided as **Supplementary Materials** (**Supplementary Figure 1**).

**Figure 1:**
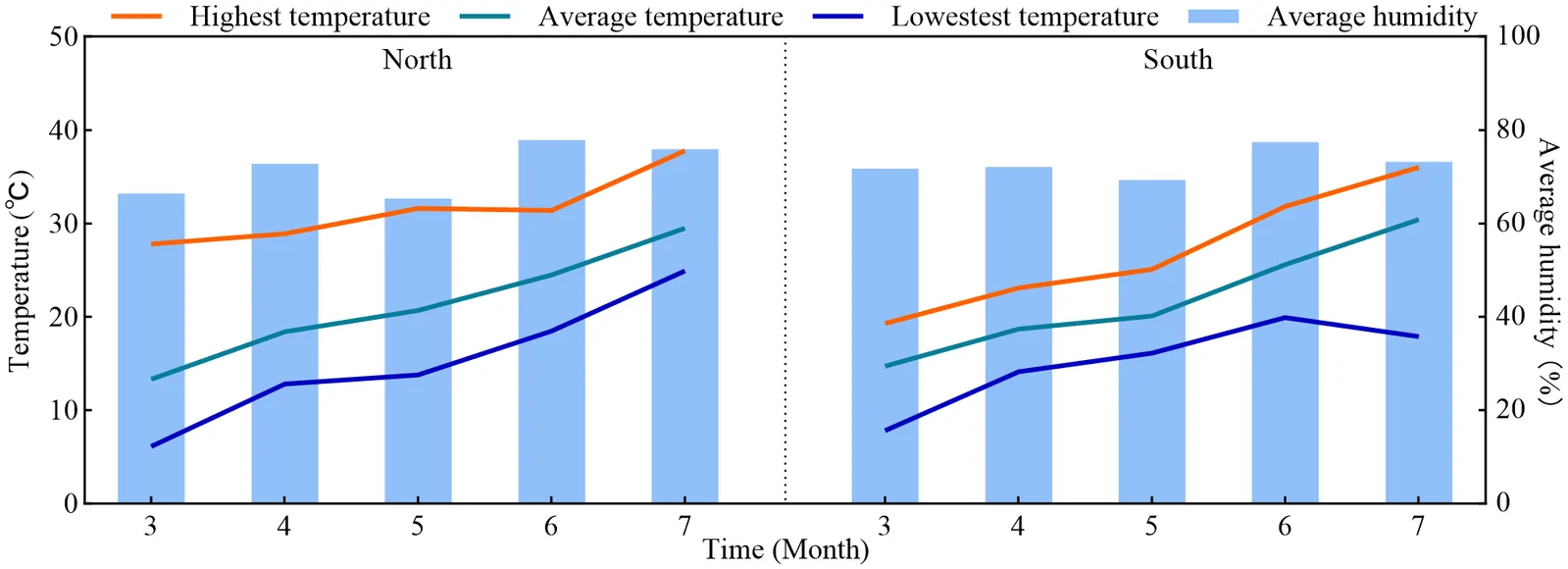
Comparison of air temperature and relative humidity between the northern and southern experimental areas during the experimental period.

**Figure 2:**
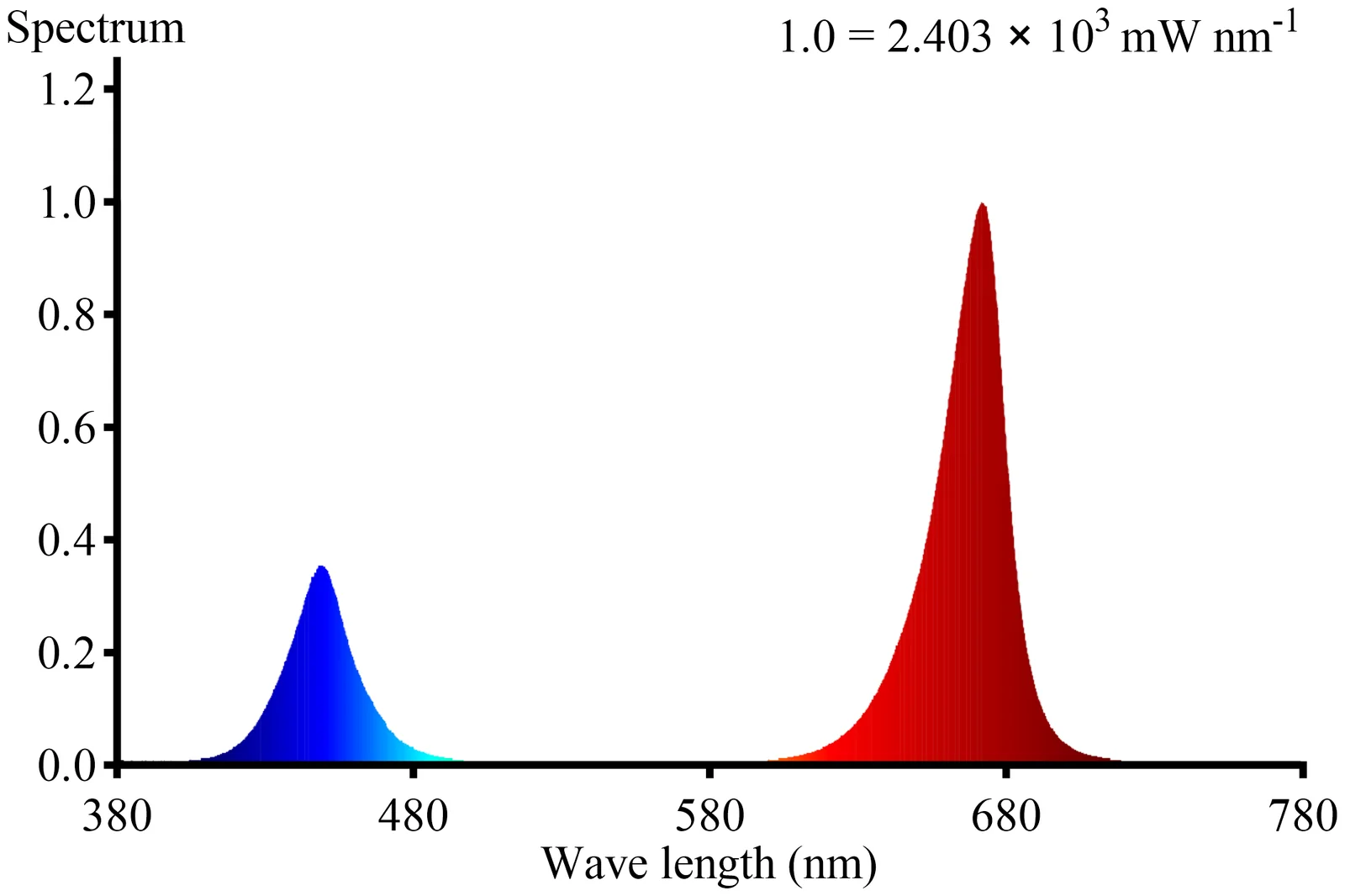
The spectral distribution of LED lighting equipment.

During the experiment, air temperature and relative humidity were continuously monitored in both areas to assess microclimatic differences associated with the lighting treatments (**Figure 1**). The turf modules and LED lighting equipment all were designed and manufactured by Beijing Teamrun Technology Development Co., Ltd. The light spectral composition for LED treatments was characterized by a consistent red (660 nm) to blue (450 nm) photon flux ratio of 7:1 (**Figure 2**). The LED fixtures were mounted 2 m above the turf canopy surface. Turf canopy level photosynthetic photon flux density (PPFD) was measured at nine points across each LED module using a series of precise and rapid spectral radiometers (Binjiang District, Hangzhou City, Zhejiang Province, China), yielding a mean PPFD of 483.67 μmol m^–2^ s^–1^. The spatial uniformity of LED illumination across the module area, expressed as the coefficient of variation (CV) of PPFD measurements, was 8.3%. The daily light integral (DLI) for the LED treatment was 20.89 mol m^–2^ d^–1^. A plant lighting analyzer (OHSP-350, Hopoocolor, Hangzhou City, Zhejiang Province, China) was employed to continuously monitored turf canopy light PPFD and spectrum for the natural-light (mean 1323.46 μmol m^–2^ s^–1^) and Shade treatments (mean 68.28 μmol m^–2^ s^–1^). The DLI range for the natural-light and Shade treatments were 15–20 mol m^–2^ d^–1^ and 0–4 mol m^–2^ d^–1^ during experimental period, respectively. The light spectral compositions for Natural-light and Shade treatments were provided as **Supplementary Materials** (**Supplementary Figure 2**).

### 2.3 Indicators measurement

Turf normalized difference vegetation index (NDVI) was measured using a turf color tester (Field Scout TCM500, Spectrum Technologies, USA). Plant samples were randomly collected using a soil sampler with a diameter of 3.5 cm and a length of 20 cm. The leaves and root systems were separated and washed clean. Roots were carefully washed with distilled water and scanned using a flatbed scanner (Epson Perfection V850 Pro, Epson, China). Root morphological parameters, including total root length and total root surface area were analyzed using the WinRHIZO root analysis system (Regent Instruments, Canada). Root dry mass was determined by oven drying at 65 °C to constant weight and expressed as g per sample. Dry weight was measured using an analytical balance with 0.1 mg precision. Specific root length (SRL) and specific root surface area (SRA) were calculated as the ratios of total root length and total root surface area to the corresponding root dry mass, respectively. Leaves were used to determine chlorophyll content, relative electrical conductivity (REC), antioxidant enzyme activity and malondialdehyde content. Fresh leaf samples (0.05–0.08 g) were cut into small pieces and placed in a 10 mL centrifuge tube, to which 8 mL of 95 % ethanol was added. The tubes were wrapped in tin foil and incubated in the dark for 48 h. Absorbance was then measured at 665, 649, and 470 nm, and total chlorophyll contents were calculated according to Arnon (Arnon, 1949). The leaves were immersed in deionized water and shaken for 24 hours. The initial conductivity was measured using a conductivity meter (DDSJ-319L, China). Then, they were placed in boiling water for 30 minutes and the conductivity was measured again. The relative conductivity was calculated based on the conductivity values before and after.

Before the determination of antioxidant enzyme activity and malondialdehyde content, fresh leaves (0.15 g) were ground in a mortar with 50 mM phosphate buffer (pH 7.0) containing 2 mM EDTA and 1% polyvinylpyrrolidone. The homogenate was centrifuged at 15000 × g for 20 min at 4 °C, and the supernatant was collected and stored in 2 mL centrifuge tubes at 4 °C for subsequent enzyme assays. Superoxide dismutase (SOD) activity was determined using the NBT photoreduction method (Kumar and Knowles, 1993), peroxidase (POD) activity by the guaiacol oxidation method (Quintanilla-Guerrero et al., 2008), and catalase (CAT) activity by hydrogen peroxide decomposition (Chance and Maehly, 1955). Malondialdehyde (MDA) content was measured using the thiobarbituric acid method (Dhindsa et al., 1981).

### 2.4 Statistical analysis

Data were analyzed using linear mixed-effects models (LMMs) to account for the repeated measurements obtained from the same turf modules across five sampling periods. Separate models were constructed for each turfgrass species and measured trait, with lighting treatment, sampling month, and their interaction included as fixed effects, while turf module identity was included as a random effect to account for the correlation among repeated observations from the same experimental units. The significance of fixed effects was evaluated using F-tests. When significant treatment effects or treatment and month interactions were detected, Tukey’s multiple comparison test was performed to determine differences among treatments within each sampling month. The turf module was considered the experimental unit, and three independent turf modules within each lighting treatment were used as biological replicates. Statistical analyses were performed using R version 4.5.1, and figures were generated using GraphPad Prism 10.0 (GraphPad Software, Inc., Calif., USA). Principal component analysis (PCA) was conducted using replicate-level observations from all sampling dates. The analysis included measurements from both turfgrass species to characterize overall multivariate response patterns under different light environments. Correlation analysis was conducted separately for each turfgrass species using treatment-by-time mean values.

## 3 Results

### 3.1 Turf appearance and chlorophyll content

**Figure 3:**
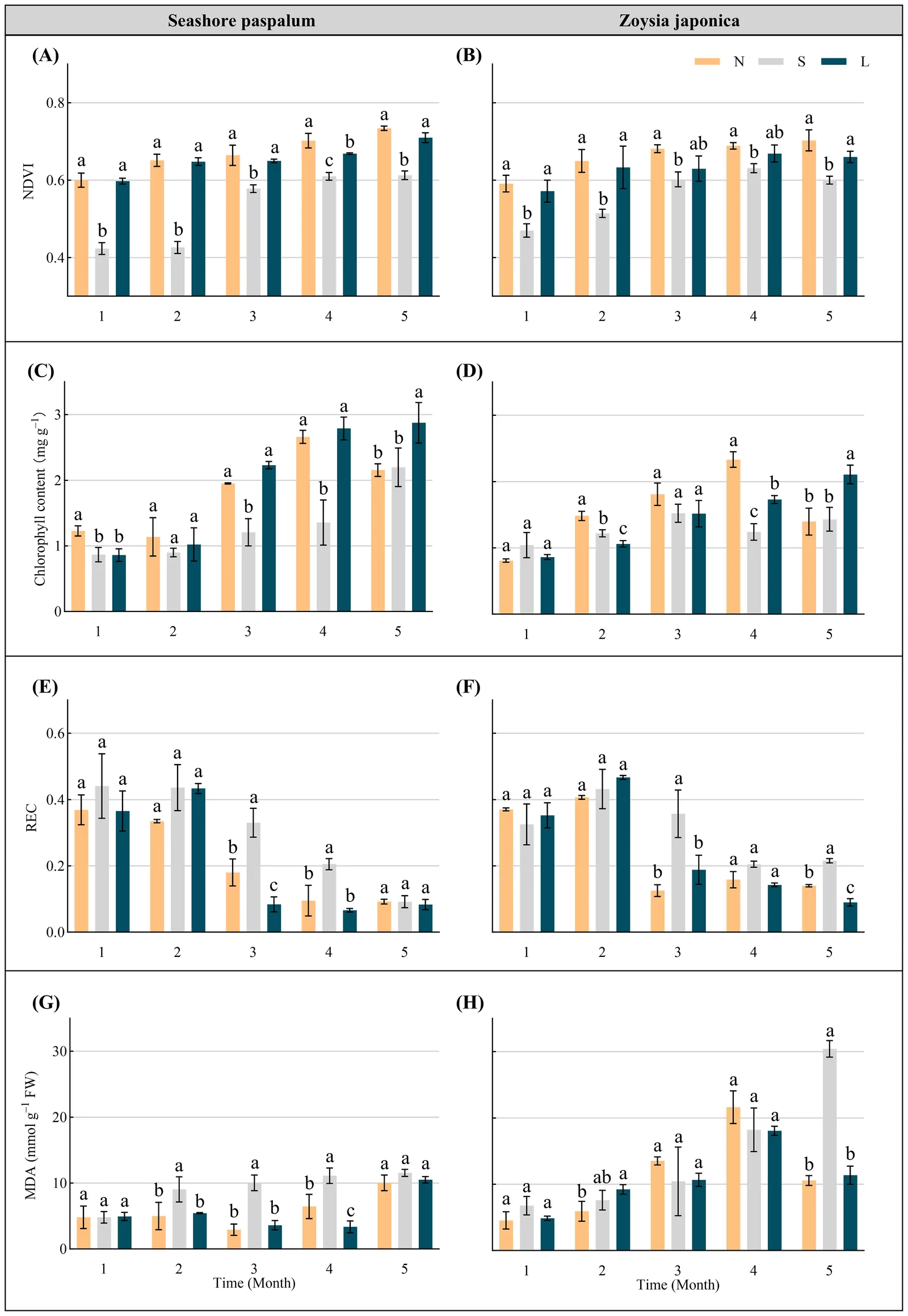
Effects of different light treatments on physiological responses of *Seashore paspalum* and *Zoysia japonica*, including normalized difference vegetation index [NDVI, **(A, B)**], chlorophyll content **(C, D)**, relative electrical conductivity [REC, **(E, F)**], and malondialdehyde [MDA, **(G, H)**] content. “N”, “S”, and “L” represent natural light, shading, and LED light treatments, respectively. Values represent means ± SD (n = 3). Different lowercase letters indicate significant differences among light treatments within the same sampling month based on Tukey’s multiple comparison test after linear mixed-effects model analysis (*p* < 0.05).

Regarding turf appearance, NDVI values of both *Seashore paspalum* and *Zoysia japonica* showed overall increasing trends over time across all treatments (**Figures 3A, B**). Under natural light, the turf consistently exhibited higher NDVI than that under shaded or LED conditions, though the difference between natural light and LED treatment was not consistently significant (**Figures 3A, B**). Meanwhile, turf subjected to LED treatment consistently maintained significantly higher NDVI values than shaded turf throughout the experimental period, particularly during the early growth stage (*p* < 0.05, **Figures 3A, B**). These trends were consistent across both *Seashore paspalum* and *Zoysia japonica*.

Chlorophyll content in both *Seashore paspalum* and *Zoysia japonica* exhibited an overall increasing trend across all treatments over the experimental period (**Figures 3C, D**). Under natural light, chlorophyll content initially increased but subsequently declined. In contrast, chlorophyll levels under LED lighting showed a consistent upward trend throughout (**Figures 3C, D**). This pattern was observed in both tested turfgrass species. However, under shade treatment, chlorophyll content in *Seashore paspalum* increased gradually, whereas in *Zoysia japonica* it rose slowly and then remained relatively stable (**Figures 3C, D**). During the early experimental phase, chlorophyll content in *Seashore paspalum* remained stable under all light treatments, followed by a mid-phase increase (**Figure 3C**). This increase was more pronounced under natural light and LED light treatment than under shade treatment (**Figure 3C**). However, chlorophyll content increased significantly during the early phase under both natural light and LED light treatment in *Zoysia japonica* (**Figure 3D**). Furthermore, chlorophyll content under natural light treatment eventually showed no significant difference compared to the shade treatment (**Figure 3D**). Meanwhile, chlorophyll content under LED light was significantly higher than under both natural light and shade treatments. This result was consistent across both *Seashore paspalum* and *Zoysia japonica* (*p* < 0.05, **Figures 3C, D**).

### 3.2 Leaf membrane integrity and oxidative damage

Leaf REC of both *Seashore paspalum* and *Zoysia japonica* exhibited an overall significant decreasing trend throughout the entire experimental period under different light treatments (**Figures 3E, F**). During the early phase, REC of *Seashore paspalum* remained relatively stable (**Figure 3E**). In contrast, REC of *Zoysia japonica* showed a brief increasing trend initially (**Figure 3F**). By the mid-experimental phase, REC of both turfgrass species decreased significantly and subsequently stabilized (**Figures 3E, F**). The decline was more pronounced under natural light and LED treatments than under shade treatment. Furthermore, under shaded conditions, REC of *Seashore paspalum* continued to decline during the mid and late experimental phases, whereas that of *Zoysia japonica* stabilized after an initial decrease (**Figure 3F**). During the same periods, REC under shade treatment was generally higher than that under natural light or LED light treatment (**Figures 3E, F**). These changing patterns were largely consistent between *Seashore paspalum* and *Zoysia japonica* (**Figures 3E, F**).

Throughout the entire experimental period, MDA content of both *Seashore paspalum* and *Zoysia japonica* exhibited an overall increasing trend (**Figures 3G, H**). MDA content was significantly lower under the LED treatment than under the shade treatment for both species, with the LED treatment showing values closer to the reference natural-light treatment (*p <* 0.05, **Figures 3G, H**). Specifically, under natural light treatment, MDA content in *Seashore paspalum* leaves remained relatively stable during the early phase but increased obviously in the mid-to-late phases (**Figure 3G**). MDA content in *Seashore paspalum* remained relatively stable under LED light until an obvious rise was observed in the later phase (**Figure 3G**). In contrast, MDA content in *Seashore paspalum* increased significantly during the early phase and thereafter remained consistently high under shade treatment (**Figure 3G**). Compared with *Seashore paspalum*, *Zoysia japonica* showed greater variability in MDA content across different light treatments (**Figure 3H**). Under both natural light and LED light treatments, MDA content in *Zoysia japonica* leaves increased steadily before declining significantly toward the end (**Figure 3H**). However, MDA content in *Zoysia japonica* leaves showed a continuously increasing trend throughout the experimental period (**Figure 3H**).

### 3.3 Activities of key antioxidant enzymes

**Figure 4:**
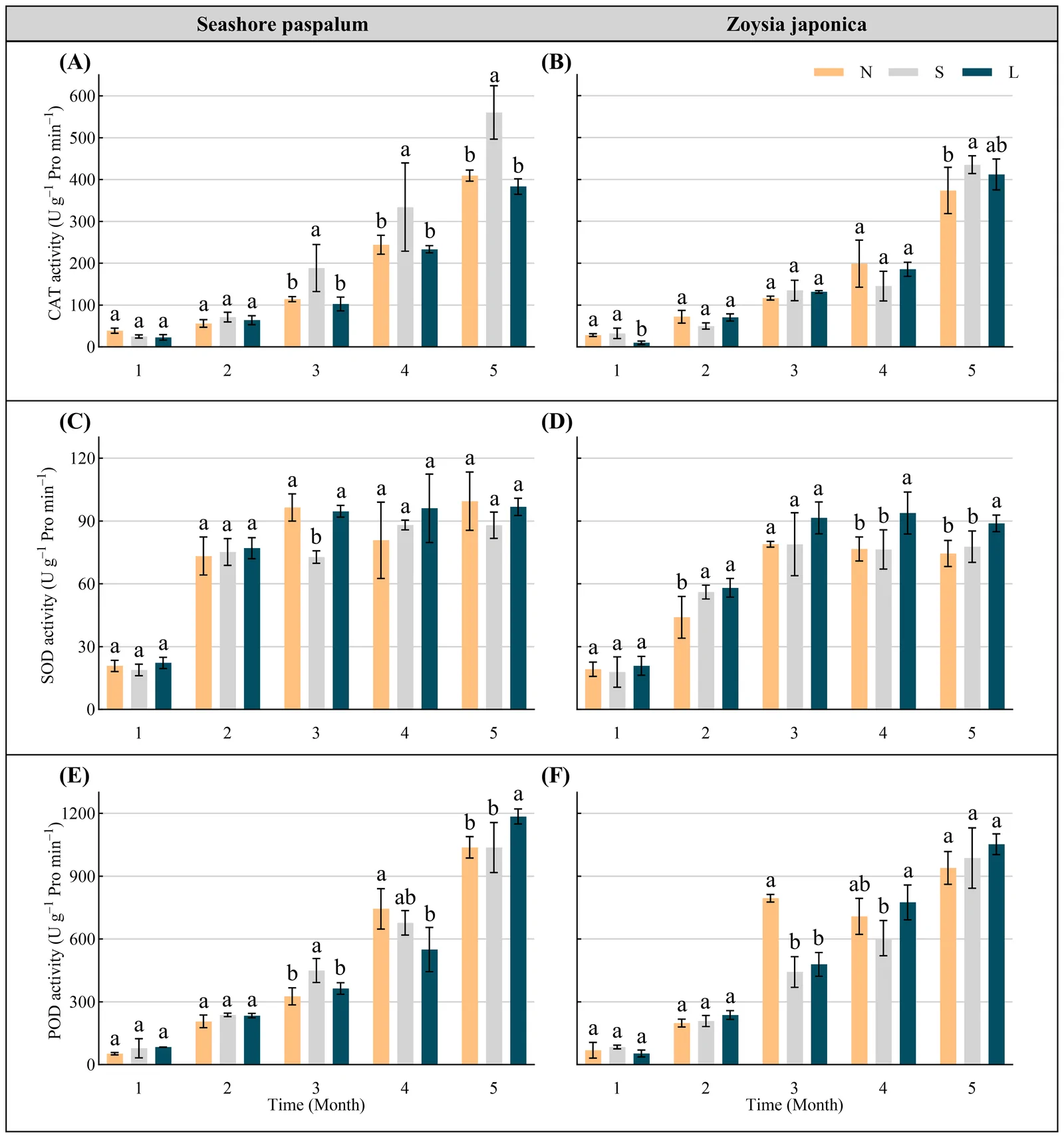
Effects of different light treatments on antioxidant enzyme activities, including catalase [CAT, (**A**, **B**)], superoxide dismutase [SOD, (**C**, **D**)], and peroxidase [POD, (**E**, **F**)], in leaves of *Seashore paspalum* and *Zoysia japonica*. “N”, “S”, and “L” represent natural light, shading, and LED light treatments, respectively. Data are presented as means ± SD (n = 3). Different lowercase letters indicate significant differences among light treatments within the same sampling month according to Tukey’s multiple comparison test following linear mixed-effects model analysis (*p* < 0.05).

Over the experimental period, the activities of antioxidant enzymes in the leaves of both *Seashore paspalum* and *Zoysia japonica* showed a significant increasing trend (**Figure 4**). For CAT activity in *Seashore paspalum*, the increase was similar under both natural light and LED light (**Figure 4A**). Moreover, CAT activity under these two treatments was consistently and significantly lower than shade treatment at the same period (**Figure 4A**). In contrast, no significant differences in CAT activity were observed in *Zoysia japonica* leaves across the different light treatments at the same period (**Figure 4B**). Regarding SOD activity, a sharp increase (nearly 3 folds) was observed in *Seashore paspalum* over a short period, after which activity remained elevated (**Figure 4C**). SOD activity was significantly higher under the LED treatment compared to the shade treatment for both species at most sampling dates (*p <* 0.05, **Figures 4A, B**). Although *Zoysia japonica* also exhibited a significant upward trend in SOD activity during the early phase, the increase was more gradual and prolonged compared to *Seashore paspalum* (**Figures 4C, D**). Furthermore, SOD activity in *Zoysia japonica* under natural light and shade was comparable and markedly lower than that under LED light (**Figure 4D**). POD activity in both grass species increased steadily over time across different light treatments (**Figures 4E, F**). However, differences in POD activity among treatments were unstable during the same periods (**Figures 4E, F**). By the late experimental phase, the highest POD activity was recorded under LED light for both species (**Figures 4E, F**). But this difference was not consistently statistically significant compared to the other treatments (**Figures 4E, F**).

### 3.4 Root morphology characteristics

**Figure 5:**
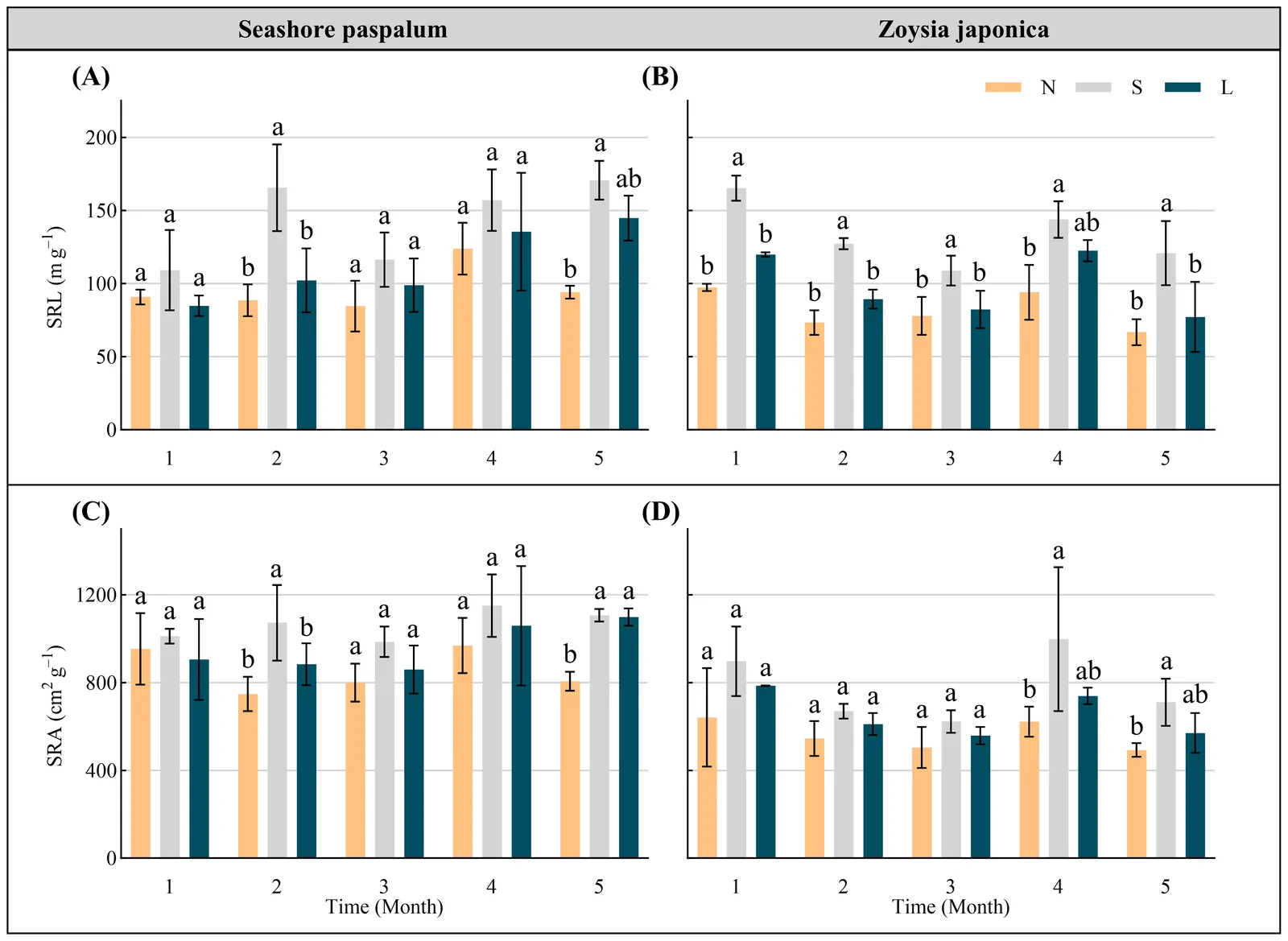
Effects of different light treatments on root morphological traits, including specific root length [SRL, (**A**, **B**)] and specific root surface area [SRA, (**C**, **D**)], of *Seashore paspalum* and *Zoysia japonica*. “N”, “S”, and “L” represent natural light, shading, and LED light treatments, respectively. Data are presented as means ± SD (n = 3). Different lowercase letters indicate significant differences among light treatments within the same sampling month according to Tukey’s multiple comparison test following linear mixed-effects model analysis (*p* < 0.05).

Under different light treatments, *Seashore paspalum* and *Zoysia japonica* exhibited contrasting trends in root morphology (**Figure 5**). Overall, both SRL and SRA tended to increase in *Seashore paspalum* but decrease in *Zoysia japonica* by the end of the experiment, although these differences were not always statistically significant (**Figure 5**). Specifically, the highest values for both SRL and SRA were recorded under shaded treatment, while the lowest values were observed under natural light (**Figure 5**). In *Seashore paspalum*, SRL remained highest under shade, decreased significantly under LED light, and was maintained at the lowest level under natural light (**Figure 5A**). Over time, the SRL of *Seashore paspalum* showed some fluctuation across treatments, yet the relative differences among light treatments remained stable (**Figure 5A**). Moreover, a similar pattern was observed for SRA, which closely mirrored the variations in SRL (**Figure 5**). For *Zoysia japonica*, the trends in SRL and SRA across light treatments were nearly identical (**Figures 5B–D**). Although values in all treatment groups fluctuated the hierarchical order among treatments (S > L > N) remained consistent throughout the experimental period.

### 3.5 Specific responses of turf to different lighting conditions

**Figure 6:**
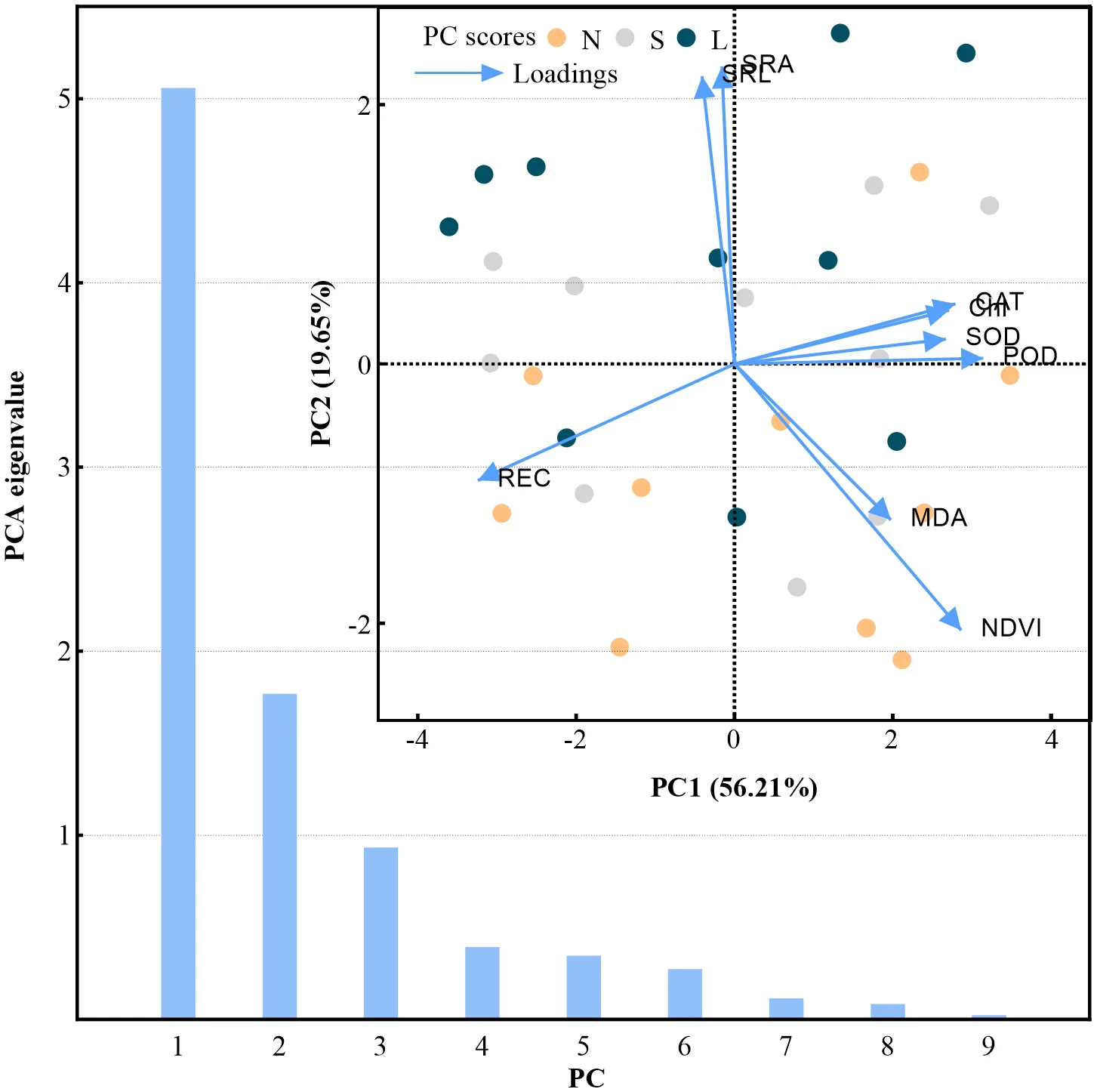
Principal component analysis (PCA) results of all measured indicators. “N” means natural light treatment, “S” means shading treatment, “L” means LED light treatment. “PC1” and “PC2” mean first and second principal component. NDVI, normalized difference vegetation index, Chl, chlorophyll content, REC, relative electrical conductivity, MDA, malondialdehyde content, SOD, superoxide dismutase activity, POD, peroxidase activity, CAT, catalase activity, SRL, specific root length and SRA, specific root surface area.

To elucidate the intrinsic relationships and variation structures among turf growth and physiological indicators under different light conditions, PCA was performed using replicate-level observations of all measured indicators. The results showed that the eigenvalues of PC1 and PC2 both exceeded 1, while the others all fell below 1, confirming the dominant role of the first two principal components (**Figure 6**). Further analysis indicated that PC1 (56.21%) and PC2 (19.65%) together accounted for 75.86% of the total variance, effectively represented the core information of the original dataset (**Figure 6**). The direction and magnitude of the loading vectors further clarified the contribution and association of each indicator with the principal components. PC1 was strongly positively correlated with NDVI, MDA, and antioxidant enzyme activities (POD, SOD, and CAT), and negatively correlated with REC, indicating that these variables contributed substantially to the variation represented by PC1. PC2 was positively associated with root morphology indicators such as SRL and SRA. Moreover, NDVI, MDA, and the antioxidant enzymes exhibited longer loading vectors, indicating their relatively greater contributions to the separation among treatments in the PCA space. Moreover, the score plots displayed clear spatial separation among the three light treatments (N, S, L), reflecting the differences in growth and physiological characteristics among groups (**Figure 6**). Specifically, the score points of the LED treatment showed distinct positioning in relation to root morphological traits in the PCA space (**Figure 6**). This indicates that LED treatment was associated with differences in root morphological traits in the PCA space. In contrast, the shaded treatment clustered toward the high-loading vectors of MDA, POD, SOD and CAT (**Figure 6**). Meanwhile, the natural-light treatment was positioned opposite to the high-loading direction of REC and toward the lower-loading direction of NDVI (**Figure 6**).

**Figure 7:**
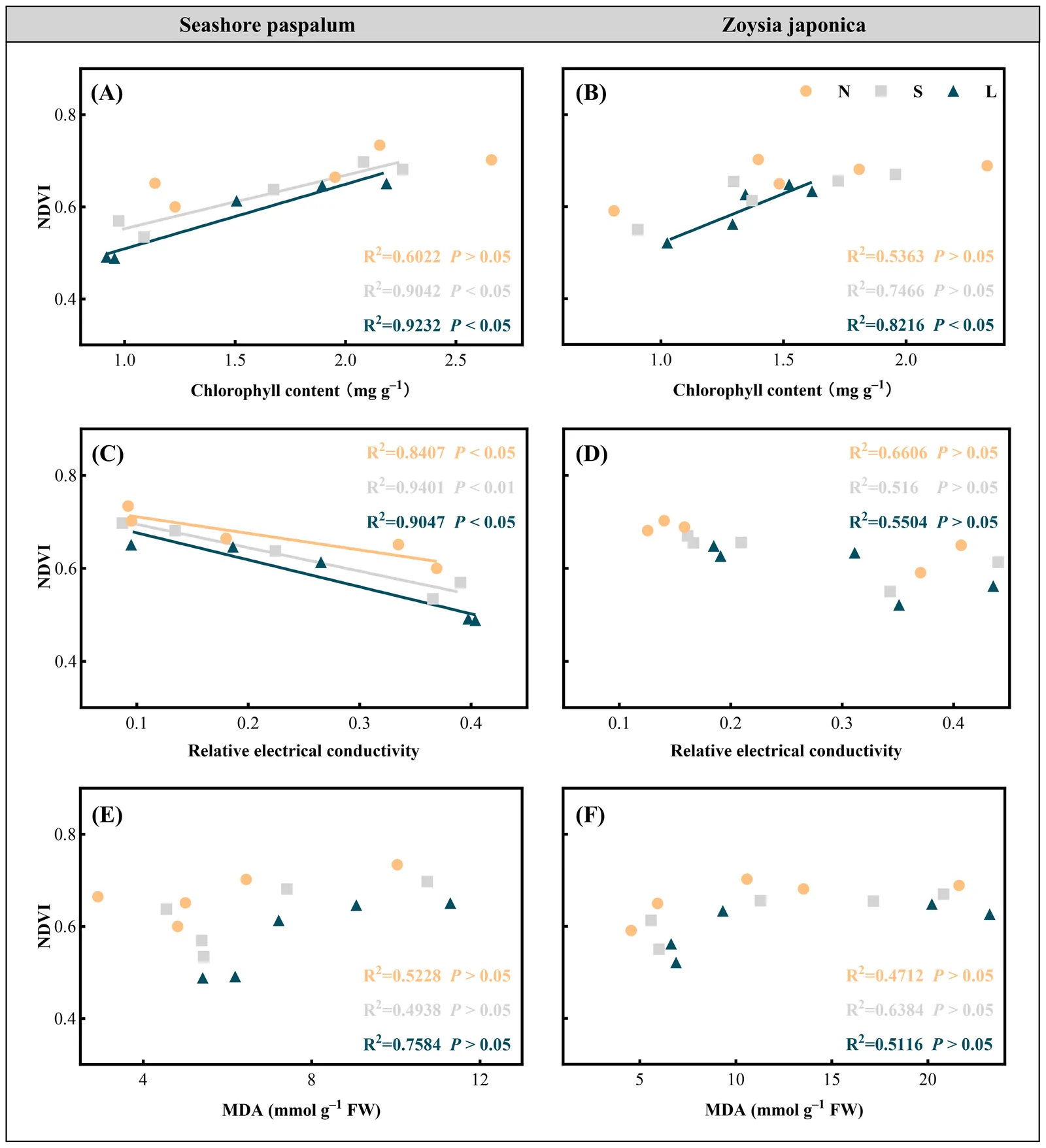
The correlation between NDVI and chlorophyll content **(A, B)**, REC **(C, D)** and MDA content **(E, F)** in *Seashore paspalum* and *Zoysia japonica*. “N” means natural light treatment, “S” means shading treatment, “L” means LED light treatment. Differences were considered statistically significant at *P* < 0.05.

**Figure 8:**
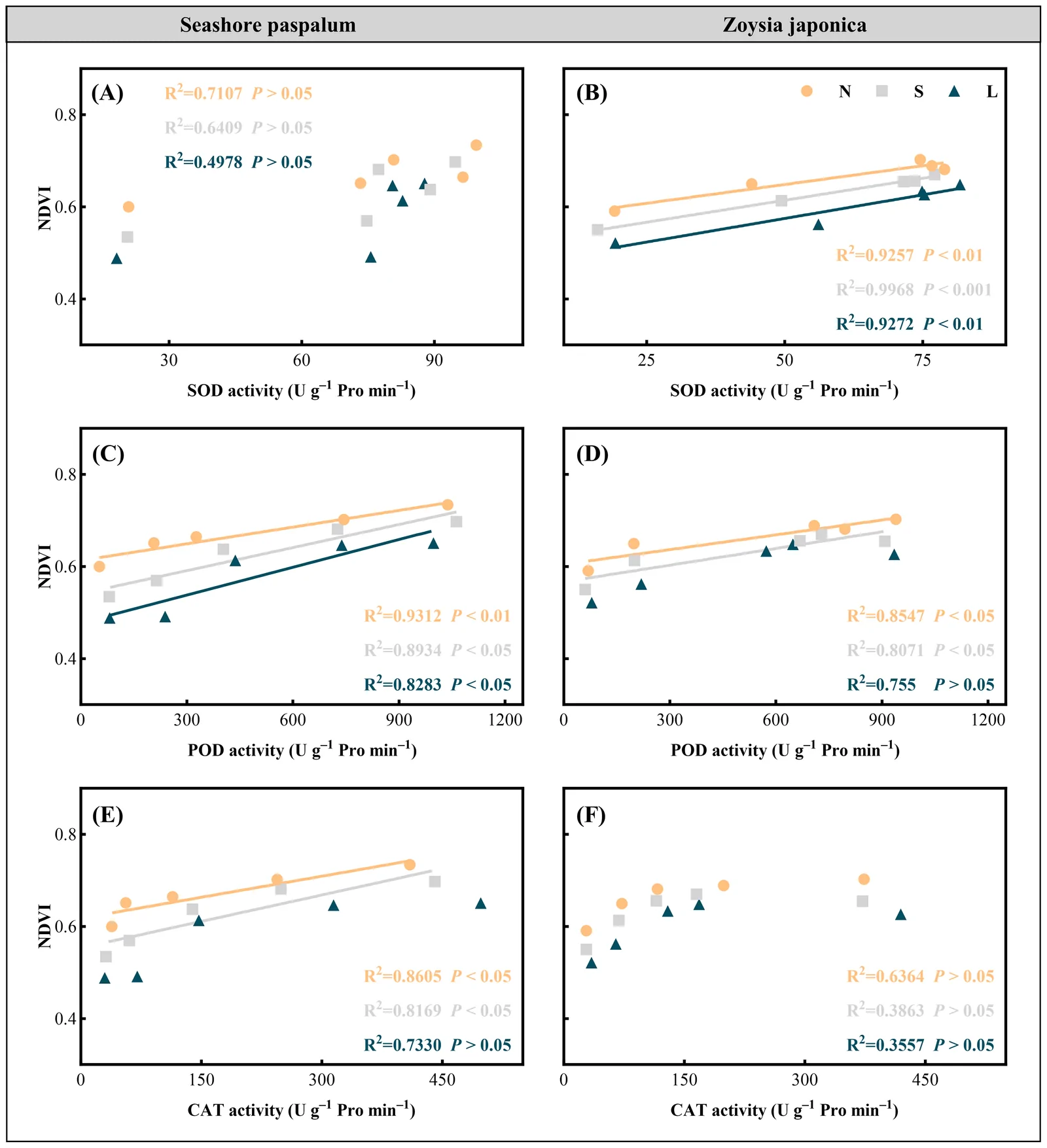
The correlation between NDVI and activities of SOD **(A, B)**, POD **(C, D)** and CAT **(E, F)** in *Seashore paspalum* and *Zoysia japonica*. “N” means natural light treatment, “S” means shading treatment, “L” means LED light treatment. SRL means specific root length, and SRA means specific root surface area. Differences were considered statistically significant at *P* < 0.05.

Correlation analyses were performed separately for each species. Turf NDVI showed significant associations with chlorophyll content, REC, and antioxidant enzyme activity during the experimental period (**Figures 7**, **8**). These relationships exhibited specificity across different light treatments. Specifically, under the various light conditions, turf NDVI showed a significant negative correlation with REC and a significant positive correlation with antioxidant enzyme activity (**Figure 7**). Additionally, a significant positive correlation was observed between NDVI and chlorophyll content under both shaded and LED treatments (**Figure 7**). Furthermore, the two turfgrass species exhibited distinct specificity in these relationships (**Figure 7**).

**Figure 9:**
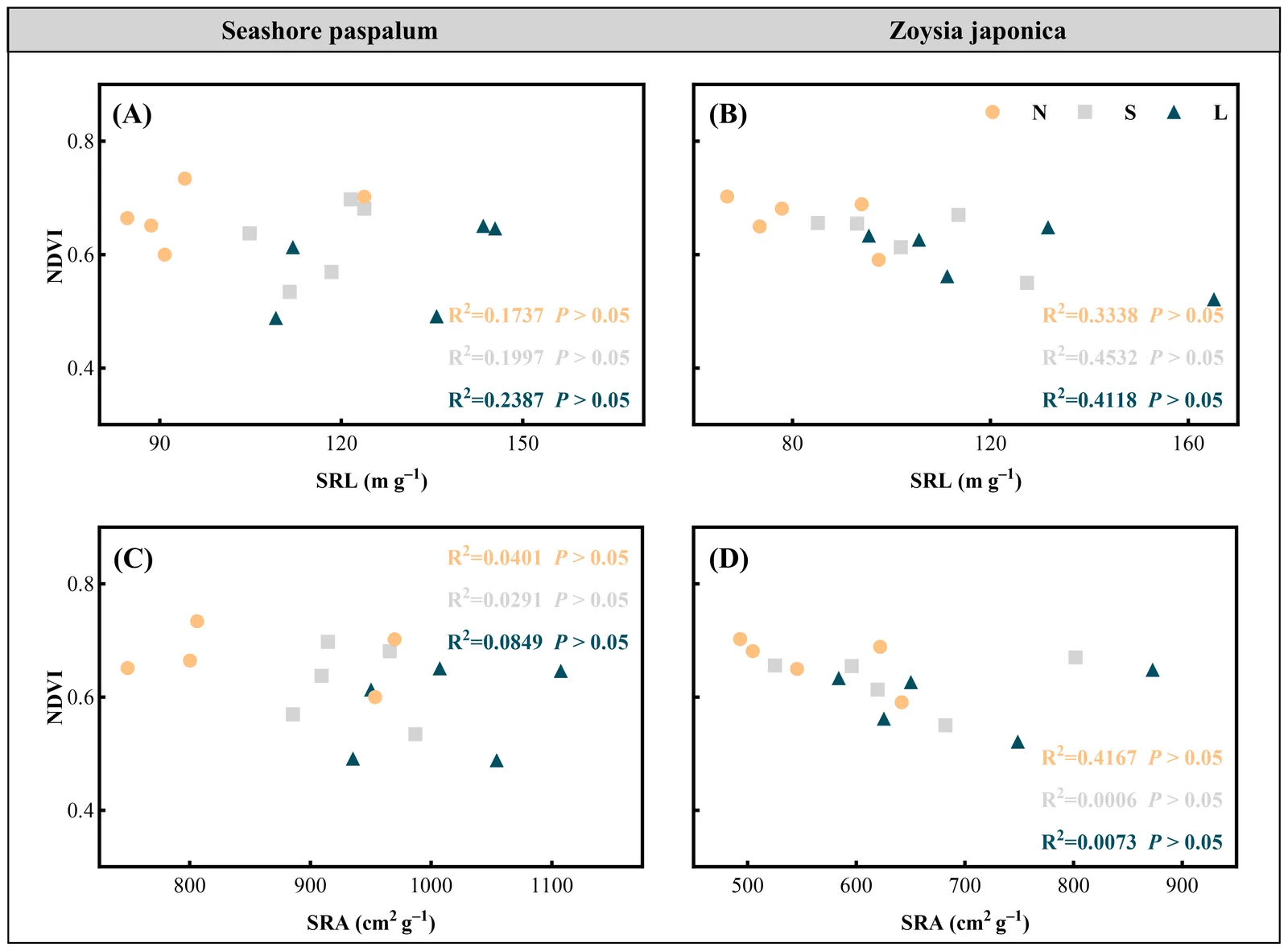
The correlation between NDVI and SRL **(A, B)** and SRA **(C, D)** in *Seashore paspalum* and *Zoysia japonica*. “N” means natural light treatment, “S” means shading treatment, “L” means LED light treatment. Differences were considered statistically significant at *P* < 0.05.

For *Seashore paspalum*, under natural light, NDVI was significantly correlated with REC, POD, and CAT activity (**Figures 7**, **8**). Under shade, NDVI was significantly correlated with chlorophyll content, REC, POD and CAT activities (**Figures 7**, **8**). Under LED treatment, NDVI showed significant correlations with chlorophyll content, REC, and POD activity (**Figures 7**, **8**). In contrast, for *Zoysia japonica*, under both natural light and shade treatments, NDVI was only significantly correlated with SOD and POD activities (**Figures 8B, D**). Under LED treatment, NDVI was only significantly positively correlated with chlorophyll content and SOD activity (**Figure 8B**). Moreover, no significant correlations were found between root morphological traits and turf NDVI across the different light conditions (**Figure 9**).

## 4 Discussion

### 4.1 Responses of turf quality and chlorophyll status to different light conditions

LED light treatment improved turf NDVI and chlorophyll content under shade conditions, approaching the levels observed under natural light (**Figure 3**). The NDVI is a commonly used metric of exposure to trees and plants, which is seen as an objective indictor to evaluate visual quality of turfgrass (Bremer et al., 2011; Donovan et al., 2022). The results showed that the turf NDVI under different light treatments exhibited an overall increasing trend over time (**Figure 3**). This suggested that seasonal environmental changes during the experimental period were associated with increased turf NDVI, while natural light and LED treatments may have contributed to the maintenance of turf appearance. However, the differences in NDVI between the natural light and LED treatments were not entirely statistically significant for either species, suggesting that the effect of LED light on turf quality was comparable to that of natural light (**Figure 3**). In addition, the seasonal increase in duration and intensity of natural light also contributed to accumulated temperature, which is more favorable for the recuperative growth of warm-season turfgrasses. In contrast, LED light is a cold light source, and its radiation does not significantly increase turf canopy temperature (Zhang et al., 2021). This may explain why the NDVI under LED light was slightly lower than that under natural light.

Chlorophyll content measurements showed that both warm-season turfgrass species gradually recovered with seasonal changes, which was associated with improved turf recovery (**Figure 3**). However, under natural light, chlorophyll content increased significantly initially but declined sharply later in the experiment (**Figure 3**). This phenomenon may be attributed to the fact that under long-term natural light conditions, solar radiation accumulates both in terms of light and temperature. As a result, there is a heat accumulation on the north side, potentially imposing additional thermal stress on turfgrass, thereby affecting chlorophyll maintenance and overall turf physiological status (Kotkowiak et al., 2017; Li et al., 2020). In contrast, turf under shaded and LED treatments experienced lower exposure to direct solar radiation during periods of high temperature and maintained relatively stable chlorophyll content. Moreover, chlorophyll content under both natural light and LED light remained consistently higher than under shade throughout the experiment. This suggested that LED supplementation can partially compensate for reduced light availability and maintain turf physiological status under shaded conditions. Although both *Seashore paspalum* and *Zoysia japonica* had been reported to exhibit good tolerance to shade environments (Jiang et al., 2004; Xu et al., 2022). The differences in NDVI and chlorophyll content between species suggested that *Seashore paspalum* and *Zoysia japonica* exhibited different response patterns to variations in light conditions.

### 4.2 Leaf membrane integrity and oxidative damage responses

The results of this study indicated that the stability of the cell membrane system of turfgrass leaves was improved under different light environments under different light environments (**Figure 3**). Compared with natural light and LED light treatments, the REC under shade showed a less pronounced decrease and remained consistently higher throughout, suggesting that low-light conditions were associated with higher membrane stability values, although the underlying mechanisms require further investigation (**Figure 3**). Low-light environment also reduced stomatal conductance and photosynthetic efficiency of plant leaf stomates (Liu et al., 2014). These results suggested that supplemental LED lighting alleviated membrane instability and oxidative stress associated with shaded conditions, which may have contributed to the observed improvements in turf physiological indicators under limited light availability.

The changing trend of MDA content revealed different oxidative stress patterns under different light environments (**Figure 3**). Under both natural light and LED light, MDA content increased significantly during the mid-to-late experimental phases, indicating increased lipid peroxidation and oxidative damage during the later experimental stages. This shift might be attributed to seasonal changes, particularly the frequent occurrence of extreme high temperatures. Because high temperatures could damage plant bio-membrane structures, reduce cellular water potential, increase the relative permeability of lipid membranes and electrolyte leakage, thereby potentially leading to physiological metabolic dysfunction in plants (Dat et al., 1998). However, under shaded conditions, the MDA content in turf leaves increased early in the experimental period and remained elevated. This suggested that low-light conditions may have altered physiological metabolism and contributed to oxidative imbalance under prolonged shade stress, leading to continuous production of reactive oxygen and consequent membrane lipid peroxidation damage (Li et al., 2025). These results highlighted that the physiological stress exerted by shaded environments on turfgrass was persistent and profound.

In conclusion, LED supplementation showed comparable effects to natural light in maintaining membrane stability and reducing oxidative damage under shaded conditions, suggesting its potential application for turfgrass management in shaded stadium environments.

### 4.3 Responses of antioxidant enzyme activities to different light conditions

The light conditions not only influenced the overall level of antioxidant enzyme activity, but more crucially regulated the differences among their activities. The shading treatment induced a significant increase in CAT activity (**Figure 4**). Under low-light stress, plants might enhance this specific pathway to scavenge hydrogen peroxide, potentially contributing to the regulation of reactive oxygen-related processes likely caused by energy limitation (Zhu et al., 2017). In contrast, LED light treatment was associated with increased SOD activity and variable changes in POD activity (**Figure 4**). It indicated that, compared to natural light, the spectral composition of LED may influence antioxidant-related responses under shaded conditions, as reflected by changes in antioxidant enzyme activities. These changes suggested altered antioxidant enzyme activity patterns under different light conditions. Under blue and yellow light, plants accumulated higher levels of superoxide anions and hydrogen peroxide, which in turn upregulated the activities of SOD, POD, and CAT in leaves to mitigate the detrimental effects of ROS (Yu et al., 2017). This is consistent with our results. In addition, red and far-red light irradiation had also been proven to be able to secretly promote the activity of antioxidant enzymes in plants such as pea seedlings (Lee et al., 2003; Wu et al., 2007). Moreover, the relatively higher POD and SOD activities observed under LED treatment during the late experimental phase suggested that LED supplementation was associated with enhanced antioxidant-related responses.

Collectively, various light environments influenced the oxidative stress defense strategies of turfgrass by differentially regulating the activities of key antioxidant enzymes such as CAT, SOD and POD. LED lighting was associated with altered antioxidant enzyme patterns, particularly increased SOD activity, which may contribute to improved physiological tolerance under shaded conditions. This feature meant that through artificial supplementary LED lighting, not only the insufficient light intensity be compensated for, but also LED lighting may influence antioxidant-related enzyme activities through spectral effects, which may contribute to differences in turf physiological responses under shaded conditions.

### 4.4 Responses of morphological characteristics of root systems

Plant roots acclimate to environmental conditions through structural modifications. Furthermore, specific adaptations in different root tissues regulate root metabolic demand and nutrient uptake efficiency (Rellán-Álvarez et al., 2016). Moreover, the morphological traits of herbaceous plant roots, along with their mechanical reinforcement of soil, contribute positively to soil stabilization (Hao et al., 2023). Especially in sports turf, soil stability is particularly important. Therefore, clarifying the responsive changes in the morphological characteristics of turf root systems under different lighting environments is an important foundation for providing a basis for optimizing turf management under different lighting environments. The results of this study showed that the SRL and SRA under shade remained at the highest level. This may be attributed to the fact that when light availability becomes a major limiting factor, plants may adjust root morphological traits to improve resource exploration under unfavorable conditions (Irving and Mori, 2021; Hong et al., 2023). Therefore, the increases in specific root length and specific root surface area observed under shaded conditions may reflect morphological responses associated with enhanced root exploration potential under limited light availability, as proposed in previous study (Xue and Li, 2016). However, whether these changes contribute to compensatory resource acquisition requires further investigation. This morphological plasticity represents an important response of plants to variations in resource environments. These viewpoints had also been confirmed and supported by other research results (Olschowski et al., 2016; Zhao et al., 2019).

Furthermore, we found that LED lighting treatment effectively reduced the high SRL and SRA observed under shaded condition to levels comparable to those under natural light, even lower. This pattern may reflect a shade-induced adjustment in root morphology when light availability is limited. Under such conditions, plants may modify root traits to maintain resource acquisition capacity (Poorter and Ryser, 2015; Hussain et al., 2021). This suggested that LED lighting modified shade-induced root morphological responses, resulting in intermediate SRL and SRA characteristics between shaded and natural light conditions. Previous studies have suggested that plant photoreceptors can influence root development and biomass distribution under different light environments, including that the mixture of red and blue light reduced root growth and modulated nitrogen distribution between shoots and roots (Yang et al., 2016; Izzo et al., 2020; Liang et al., 2022).

In summary, light conditions exerted a continuous and strong influence on root morphology, with their effects outweighing those of growth stage or short-term environmental variations. By alleviating light limitation, LED lighting alleviated the magnitude of shade-induced changes in root morphology. In shaded areas of professional sports stadiums, LED lighting can be regarded as an effective cultural management strategy to address the significant challenge of natural light deficiency in turf maintenance.

### 4.5 Multivariate characterization of turf responses to different light environments

This study elucidated the response patterns of *Seashore paspalum* and *Zoysia japonica* to different light environments through a combined analysis of PCA and correlation analysis, thereby providing theoretical support for precision light management in turf maintenance. The first two principal components cumulatively accounted for 75.86% of the total variance, and all subsequent eigenvalues were less than 1, which is consistent with the standard criteria for PCA validity (Gewers et al., 2022). This indicated that the selected indicators captured major sources of variation among light treatments. The obvious separation of scoring points with different lighting treatments in the PCA space indicated that shading, LED lighting and natural light environments respectively resulted in distinct physiological response patterns. Under natural light, there is a trade-off relationship between the stability of turf cell membranes and vegetation coverage. This might be related to the fact that under long-term natural light conditions, solar radiation accumulates both in terms of light and temperature (**Figure 1**). The accumulation of temperature caused by solar radiation can easily damage the integrity of plant cell membranes and photosynthetic organs, thereby causing grass to exhibit heat stress (Long et al., 1994; Adir et al., 2005; Shi et al., 2022).

In contrast, turf under shaded conditions showed increased antioxidant enzyme activities, which was closely associated with the physiological characteristic of warm-season turfgrasses requiring higher light levels (Zhang et al., 2017). Shading environment easily reduced photosynthetic efficiency and elevates MDA content. Antioxidant enzymes such as SOD, POD, and CAT help maintain cellular homeostasis by synergistically scavenging excess ROS, which are commonly involved in ROS-related regulation under stress conditions (Fu et al., 2014; Raza et al., 2020; Sun et al., 2023). However, LED lighting might influence root morphological responses under different light environments and promoting adjustments in root architecture under different light environments. For example, blue light can stimulate root initiation and growth (Gil et al., 2020; Navidad et al., 2020; Rasool et al., 2020).

Correlation analysis further revealed the light environment specificity and species-specific differences of the relationships between NDVI and physiological indicators. As a core indicator of turf growth status, NDVI showed significant correlations with cell membrane stability, antioxidant capacity, and photosynthetic pigment accumulation only under certain light environments and in specific turfgrass species. This suggested that both the light environment and grass species jointly regulated the coupling strength between NDVI and underlying physiological processes. Notably, although root morphology was identified as a core loading factor under LED lighting in the PCA, no significant correlation was observed between root morphological traits and NDVI. Turf NDVI, as an apparent indicator, mainly reflects the short-term growth status of the turf canopy (Bell et al., 2002). However, the adjustment of root morphology involves long-term or indirect adaptation processes such as carbon allocation and resource acquisition strategies (Huang and Fry, 1998; Lee et al., 2017). Therefore, there was not necessarily a simple linear relationship between turf NDVI and root morphology. In other words, plant aboveground and belowground parts exhibit relative independence in their environmental responses (Zhang et al., 2019; Liu et al., 2024). Therefore, when evaluating the overall impact of light environment on plants, it is essential not to rely solely on apparent indicators such as NDVI while neglecting root morphology as a critical dimension. Furthermore, the differences in response patterns between *Seashore paspalum* and *Zoysia japonica* underscore the importance of varietal selection in light-adaptation management for sports turf. In summary, when evaluating the overall effect of light environments on plants, it is essential not to rely solely on apparent indicators such as NDVI while neglecting root morphology as a critical dimension. Furthermore, the differences in response patterns between both turfgrass species underscore the importance of species selection in light-adaptation management for sports turf.

Because LED supplementation was applied under a realistic stadium environment, the observed responses may reflect the combined effects of increased photon availability, altered photoperiodic conditions, and potential spatial variation. Although microclimatic monitoring indicated comparable environmental conditions among locations, the influence of site-specific factors cannot be completely excluded. Moreover, this study focused primarily on turf growth and physiological responses, and further long-term field evaluations considering practical factors such as energy efficiency and performance under use conditions are required before broader application recommendations can be established.

## 5 Conclusions

This study systematically evaluated the physiological and morphological responses of *Seashore paspalum* and *Zoysia japonica* to different light environments through a real scenario simulation experiment conducted inside a professional stadium. Results indicated that the tested 7:1 red-blue LED lighting supplementation partially alleviated shade-induced reductions in *Seashore paspalum* and *Zoysia japonica* turfgrass physiological and morphological traits, including turf appearance quality, chlorophyll status, membrane stability, antioxidant responses, and root morphological characteristics. Specifically, LED lighting restored turf NDVI and chlorophyll content to levels close to those under natural light conditions, indicating improved canopy greenness and maintenance of photosynthetic pigments under shaded environments. Moreover, LED lighting was associated with improved physiological responses related to environmental stress. This was associated with improved membrane stability, reduced membrane lipid peroxidation, and altered antioxidant enzyme responses, including increased SOD activity and changes in POD activity. Regarding root morphology, LED lighting moderated the shade-induced changes in root morphological traits and altered root morphological responses under shaded conditions. PCA and correlation analyses further revealed distinct response patterns associated with different light environments. LED lighting treatment was associated with concurrent changes in aboveground physiological traits and root morphological responses under shaded conditions. This study provides experimental evidence for the application potential of supplemental LED lighting under shaded stadium conditions.

## Data Availability

The raw data supporting the conclusions of this article will be made available by the authors, without undue reservation.
