# Supplementary material for: LED lighting as a solution for turf shading: insights from a systemic assessment of Seashore paspalum and Zoysia japonica
Source: Front Plant Sci. 2026 Sep 4;17:1936506. doi: 10.3389/fpls.2026.1936506 (PMC13585661; doi:10.3389/fpls.2026.1936506)
Supplement: Supplementary file 1 [file Presentation1.pdf]

Spectrum Test Report

Sample	: LED 灯具	Date	: 2021-04-08 11:06:22
Specification	: 投光灯	Sam. Status	:
Sample No.	: 210407106001	Standard	:
Manufacturer	: 博德	Instrument	: HaasSuite(EVERFINE)
Assessor	: damin		
Remark	: ---	Test by	: Pengkang Liang
Device SN	:		

Test Condition

Temprature	: 25Deg	RH	: 65%
WL Range	: 380nm-780nm	IP	: 51004 (78%)
Test Mode	: Accuracy Test	T	: 11 ms
Sensitivity	: Low		

Spectrum

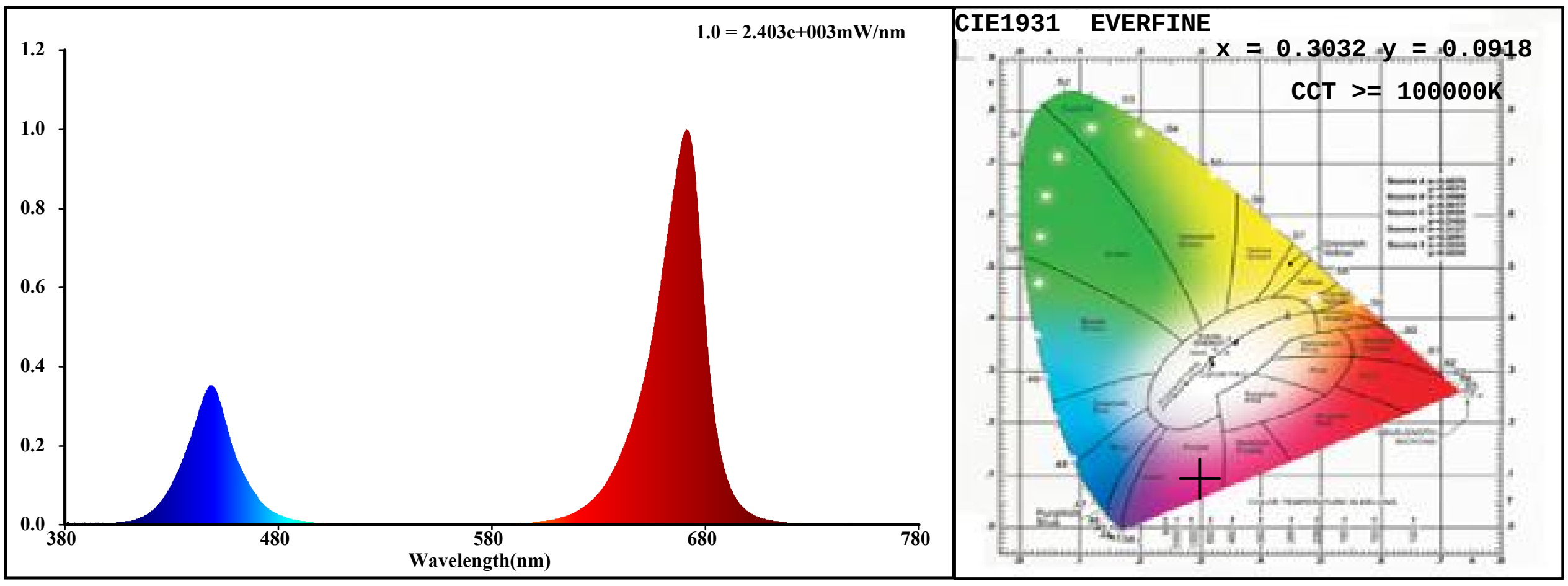

Colorimetric Parameters

Chromaticity Coordinate: x = 0.3032 y = 0.0918 / u' = 0.3470 v' = 0.2364 (duv=-1.99e-01)

CCT>=100000K Prcp WL: Ld=-559.6nm Purity=82.1%

Peak WL: Lp=671nm FWHM: =24.2nm Ratio:R=79.2% G=3.1% B=17.7%

Render Index: Ra = -267.3

EEI: 2.84770 E Lowest

R1 =-294	R2 =-376	R3 =-396	R4 =-52	R5 =-295	R6 =-366	R7 =-58	
R8 =-301	R9 =-999	R10=-960	R11=-76	R12=-632	R13=-385	R14=-127	R15=-477

WHITE:OUT

Photometric & Radiometric Parameters

Photosynthetic: PPFD:13533.37umol/s/m2 (Distance:0.5m) PPFD:3388.34umol/s/m2 (Distance:1.0m)

Photosynthetic: PPFD:1505.93umol/s/m2 (Distance:1.5m) PPFD:847.09umol/s/m2 (Distance:2.0m)

Electrical parameters

V = 220.12 V I = 3.761 A P = 821.0 W PF = 0.9918

LCTECH Guangdong Testing Services Co., Ltd.
