# Supplementary material for: LED lighting as a solution for turf shading: insights from a systemic assessment of Seashore paspalum and Zoysia japonica
Source: Front Plant Sci. 2026 Sep 4;17:1936506. doi: 10.3389/fpls.2026.1936506 (PMC13585661; doi:10.3389/fpls.2026.1936506)
Supplement: Supplementary file 2 [file Presentation2.pdf]

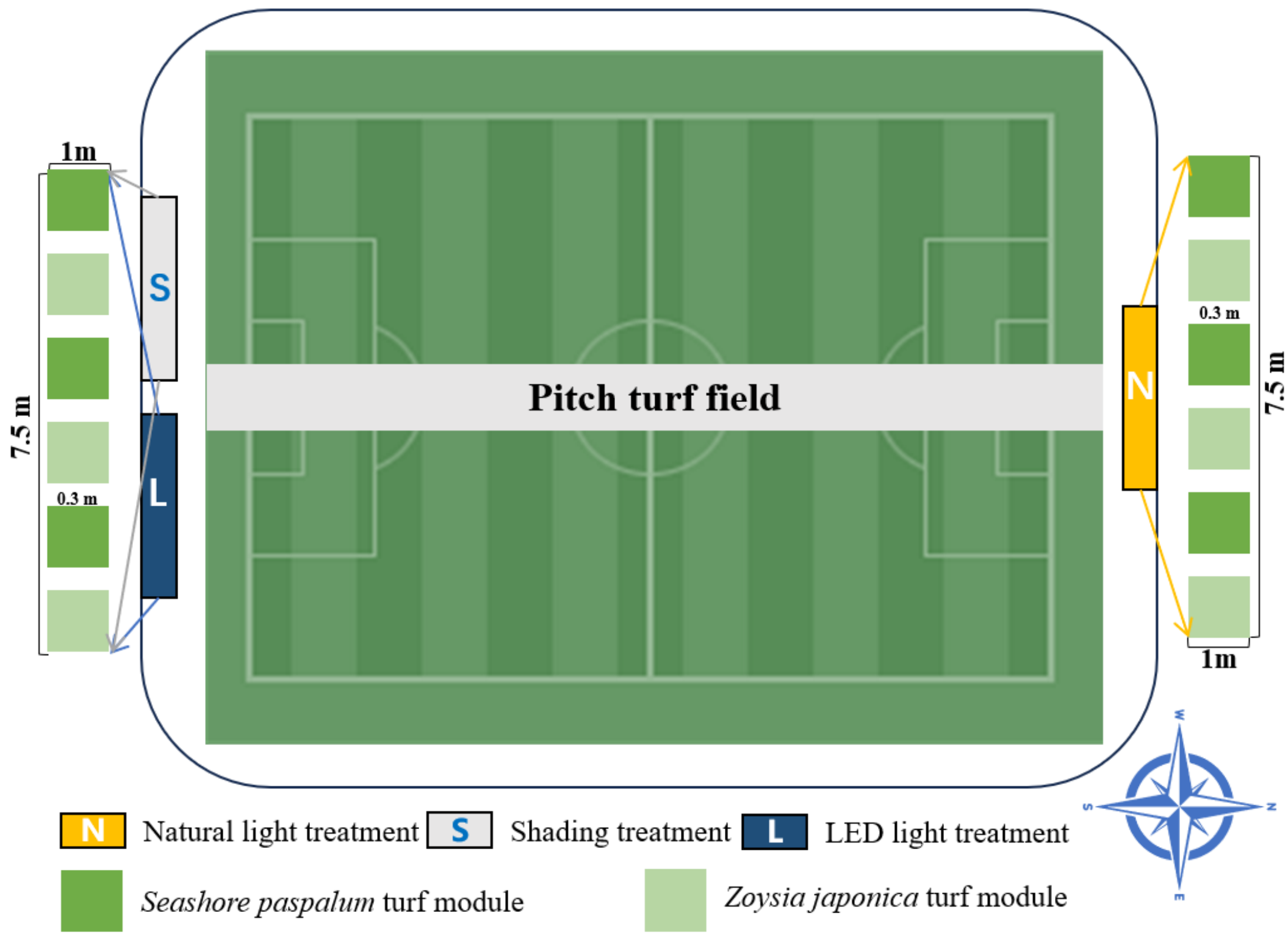

Fig. S1 Schematic diagram of the layout of experimental turf modules in the venue

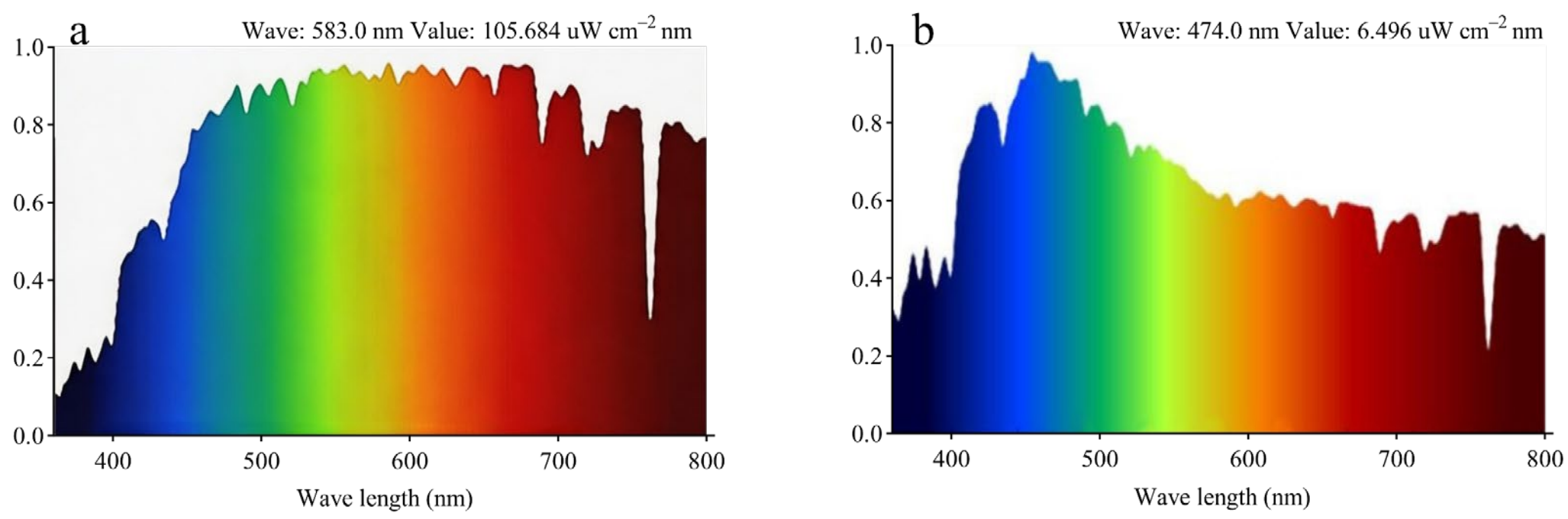

Fig. S2 The spectral distribution of Natural light treatment (a) and Shading treatment (b).
